# Long-term prognostic impact of fasting plasma glucose and myocardial flow reserve beyond other risk factors and heart disease phenotypes

**DOI:** 10.1093/ehjimp/qyae070

**Published:** 2024-07-13

**Authors:** Elena Filidei, Chiara Caselli, Luca Menichetti, Michela Poli, Debora Petroni, Letizia Guiducci, Oreste Sorace, Patrizia Pisani, Silvia Pardini, Danilo Bonora, Assuero Giorgetti, Alessia Gimelli, Danilo Neglia

**Affiliations:** Imaging Department—Nuclear Medicine Unit, Fondazione Toscana Gabriele Monasterio, Pisa, Italy; CNR Institute of Clinical Physiology (IFC), Pisa, Italy; CNR Institute of Clinical Physiology (IFC), Pisa, Italy; CNR Institute of Clinical Physiology (IFC), Pisa, Italy; CNR Institute of Clinical Physiology (IFC), Pisa, Italy; CNR Institute of Clinical Physiology (IFC), Pisa, Italy; CNR Institute of Clinical Physiology (IFC), Pisa, Italy; CNR Institute of Clinical Physiology (IFC), Pisa, Italy; CNR Institute of Clinical Physiology (IFC), Pisa, Italy; CNR Institute of Clinical Physiology (IFC), Pisa, Italy; Imaging Department—Nuclear Medicine Unit, Fondazione Toscana Gabriele Monasterio, Pisa, Italy; Imaging Department—Nuclear Medicine Unit, Fondazione Toscana Gabriele Monasterio, Pisa, Italy; Cardiovascular Department, Fondazione Toscana Gabriele Monasterio, Via G. Moruzzi 1, 56124 Pisa, Italy

**Keywords:** myocardial blood flow reserve, fasting plasma glucose, coronary microcirculation, coronary artery disease, heart failure, prognosis

## Abstract

Cardiometabolic risk factors, including high fasting plasma glucose (hFPG), are emerging prognostic determinants in patients with coronary artery disease (CAD) or heart failure (HF). Coronary microvascular dysfunction might be a comprehensive risk predictor in these patients. The purpose of this study was to assess whether hFPG and global myocardial blood flow (MBF) reserve measured by positron emission tomography (PET), expressing global coronary function, predict long-term prognosis beyond other risk factors and presence of obstructive CAD or left ventricular (LV) dysfunction associated with HF. We retrospectively collected long-term follow-up data in 103 patients (mean age 61 ± 10 years, 74 males) with stable chest pain or dyspnoea who underwent cardiac PET/computerized tomography and coronary angiography. Disease phenotypes included obstructive CAD (35%), LV dysfunction without obstructive CAD (43%), or none (22%). At multivariable logistic regression analysis, MBF reserve lower than the median value (OR 1.8, 95% CI 1.5–2.2) was significantly associated with male gender (OR 3.45, 95% CI 1.21–9.83) and hFPG (OR 3.87, 95% CI 1.17–12.84) among all risk factors. In a median follow-up of 10.9 years (interquartile range 7.8–13.9), 39 patients (37.8%) died (13.6% cardiac death). At multivariable Cox analyses including all risk factors and disease phenotypes, age (HR 1.07, 95% CI 1.02–1.12), hFPG (HR 2.18, 95% CI 1.02–4.63), and depressed MBF reserve (HR 4.47, 95% CI 1.96–10.18) were independent predictors of death (global *χ*^2^ 37.41, *P* = 0.0004). These results suggest a strong long-term prognostic role of hFPG and depressed MBF reserve in a high-risk population of patients with a high prevalence of obstructive CAD or HF.

Cardiovascular diseases (CVD) are still the major cause of death worldwide,^1^ and heart diseases including coronary artery disease (CAD) and heart failure (HF) are the most relevant.^2,3^ There is evidence of epidemiological shifts in CVD risk factors, including older age and the emerging role of cardiometabolic risk conditions mainly represented by prediabetes and type 2 diabetes.^1–3^ On the other hand, in patients with stable angina and/or equivalents the recognition of obstructive CAD is decreasing, while non-obstructive CAD and/or left ventricular (LV) dysfunction associated with HF is increasing.^4^ The possible causal connection between these evidences and the long-term prognostic implications of such changing scenarios are unclear.

In this context, coronary microvascular disease, which refers to abnormal structure and function of the coronary microcirculation, may have a relevant role. It is prevalent across a broad spectrum of CVD risk factors, may coexist with coronary atherosclerosis and LV dysfunction, and is associated with an increased risk of adverse events.^5–8^ Measurement of absolute myocardial blood flow (MBF) and MBF reserve by positron emission tomography (PET), expressing global coronary function, is a valuable tool to detect the integrated effects of risk factors, atherosclerosis, and microvascular dysfunction on the coronary circulation and to stratify related patient risk.^5–9^

The hypothesis of this retrospective study was that emerging CVD risk factors and global MBF reserve may have an independent and/or synergistic role to predict long-term prognosis in patients with stable angina and/or equivalents beyond traditional risk determinants and the heart disease phenotype.

In our institution, we retrospectively collected long-term follow-up data in patients undergoing cardiac PET/computerized tomography (CT) between 2002 and 2011 mainly because of chronic symptoms suggestive of obstructive CAD and/or HF. Among 281 consecutive patients, we selected 103 patients in whom quantitative perfusion PET data, computerized tomography coronary angiography (CTCA) or invasive coronary angiography (ICA) data, and clinical information at baseline and at prolonged follow-up were available.

At the PET/CT study, ^13^N-ammonia was used as a flow tracer to measure MBF (mL/min/g) at rest and after intravenous dipyridamole, while CT was used for attenuation correction. In 36/103 patients, a 64-slice CTCA was performed 1–6 weeks before or after the PET/CT study, while the remaining patients underwent ICA. In 19 patients, both CTCA and ICA were performed and ICA results were used to confirm the presence of obstructive CAD. Details of PET/CT and CTCA protocols and analysis have been described elsewhere.^8^

The mean age was 61 ± 10 years (74 males, 72%). Symptoms mainly included stable angina or dyspnoea (81%). Family history of CVD was present in 58%. Among established CVD risk factors, high LDL-cholesterol (hypercholesterolaemia under treatment or LDL-C > 130 mg/dL) was present in 72%, high systemic blood pressure (hypertension under treatment or SBP > 130/85 mmHg) in 51%, smoking habits in 49%, obesity defined by high BMI (BMI > 30 Kg/m^2^) in 22%, and type 2 diabetes under treatment in 17%. Among emerging cardiometabolic risk factors, low HDL-cholesterol (HDL-C < 40 mg/dL in males and <50 mg/dL in females) was present in 51%, high triglycerides (TG > 150 mg/dL) in 27%, and high fasting plasma glucose (hFPG > 100 mg/dL) in 33%. The majority of patients were under medical treatment (87%). In the whole population, global resting MBF was 0.53 ± 0.13, stress MBF 0.98 ± 0.41, and MBF reserve 1.86 ± 0.54. Global MBF reserve was <2 in 68% of patients. Obstructive CAD (>50% stenosis in at least one main vessel) was documented at CTCA and/or ICA in 35% of patients (previous myocardial infarction in 12%), while 43% had global systolic LV dysfunction without obstructive CAD (LVEF < 50% and >40% in 16%, ≤40% in 27%). Baseline clinical, PET, and coronary angiography data as well as follow-up events in the whole population and subgroups defined by heart disease phenotypes (i.e. obstructive CAD, LV dysfunction without obstructive CAD, or none) are reported in *Table 1*.

**Table 1:** Baseline clinical characteristics of the study population

	Whole population *n* = 103	No Obstr CAD No LV Dysf *n* = 21	Obstructive CAD *n* = 37	Left ventricular dysfunction *n* = 45	*P* value
**Demographic/Clinical data**					
Age, years	61 ± 10	57 ± 11	63 ± 9	61 ± 10	0.108
Sex Male	74 (72)	8 (38)	33 (89)	33 (73)	**<0**.**001**
Stable chest pain or Dyspnoea	83 (81)	19 (90)	35 (95)	29 (54)	**<0**.**001**
LVEF, %	49 ± 14	63 ± 4	56 ± 10	37 ± 9	**<0**.**001**
**Risk factors**					
Family history of CAD	62 (60)	14 (67)	25 (68)	23 (51)	0.112
Smoking	50 (49)	6 (29)	22 (59)	22 (49)	0.077
Hypercholesterolaemia	74 (72)	12 (57)	30 (81)	32 (71)	0.148
Hypertension	53 (51)	12 (57)	24 (65)	17 (38)	**0**.**043**
Obesity	23 (22)	7 (33)	6 (16)	10 (22)	0.322
Type 2 diabetes	18 (17)	2 (10)	10 (27)	6 (13)	0.150
High TG	28 (27)	5 (24)	10 (27)	13 (29)	0.911
Low HDL-C	53 (51)	11 (52)	20 (54)	22 (49)	0.893
hFPG	34 (33)	4 (19)	14 (38)	16 (36)	0.305
**PET**					
SDS (units)	3.45 ± 5.34	1.15 ± 1.57	5.72 ± 6.45	1.67 ± 4.97	**0**.**016**
Rest MBF (mL×min×g^−1^)	0.53 ± 0.13	0.63 ± 0.15	0.49 ± 0.13	0.52 ± 0.10	**<0**.**001**
Stress MBF (mL×min×g^−1^)	0.98 ± 0.41	1.37 ± 0.54	0.89 ± 0.34	0.87 ± 0.26	**<0**.**001**
MBF reserve	1.86 ± 0.54	2.19 ± 0.64	1.83 ± 0.48	1.74 ± 0.48	**0**.**005**
Depressed MBF reserve <2	70 (68)	9 (43)	27 (73)	34 (76)	**0**.**021**
**CTCA/ICA**					
Normal vessels	54 (52)	14 (67)	0 (0)	40 (89)	**<0**.**001**
Non-obstructive CAD	12 (52)	7 (33)	0 (0)	5 (11)	
Obstructive CAD	37 (34)	0 (0)	37 (100)	0 (0)	
**Follow-up**					
Coronary revascularization	17 (17)	0 (0)	14 (38)	3 (7)	**<0**.**001**
Any cause death	41 (40)	3 (14)	13 (35)	25 (56)	**0**.**005**
MACE	60 (58)	3 (14)	25 (68)	32 (71)	**<0**.**001**

Continuous variables are presented as mean ± SD and categorical variables as absolute *n* and (%). Continuous data were compared with Kruskal–Wallis test and categorical variables with *χ*^2^ test. *P* values <0.05 (bold) show statistically significant differences.

CAD, coronary artery disease; LVEF, left ventricle ejection fraction; TG, triglycerides; HDL-C, high-density lipoprotein cholesterol; hFPG, high fasting plasma glucose; PET, positron emission tomography; SDS, summed difference score; MBF, myocardial blood flow; CTCA, computerized tomography coronary angiography; ICA, invasive coronary angiography; MACE, major adverse cardiovascular events.

In a median follow-up of 10.9 years (7.8–13.9), 17 patients (17%) were revascularized (percutaneous coronary intervention, PCI 14%, CABG 3%) and 39 patients (37.8%) died (13.6% cardiac, 12.6% non-cardiac, and 11.6% unknown cause).

At multivariate logistic regression analysis, a more depressed MBF reserve, i.e. lower than the median value (OR 1.8, 95% CI 1.5–2.2), was significantly associated with male gender (OR 3.45, 95% CI 1.21–9.83) and hFPG (OR 3.87, 95% CI 1.17–12.84) among all risk factors.

At multivariable Cox analyses including all risk factors and MBF reserve, age (HR 1.06, 95% CI 1.02–1.11), hFPG (HR 2.28, 95% CI 1.09–4.79), and depressed MBF reserve (HR 4.79, 95% CI 2.14–10.69) were the only independent predictors of death (global *χ*^2^ 36.74, *P* = 0.0001) (*Table 2*). At Kaplan–Meier survival analysis, the combination of hFPG and depressed MBF reserve was associated with the worst prognosis (*Figure 1A*).

**Figure 1:**
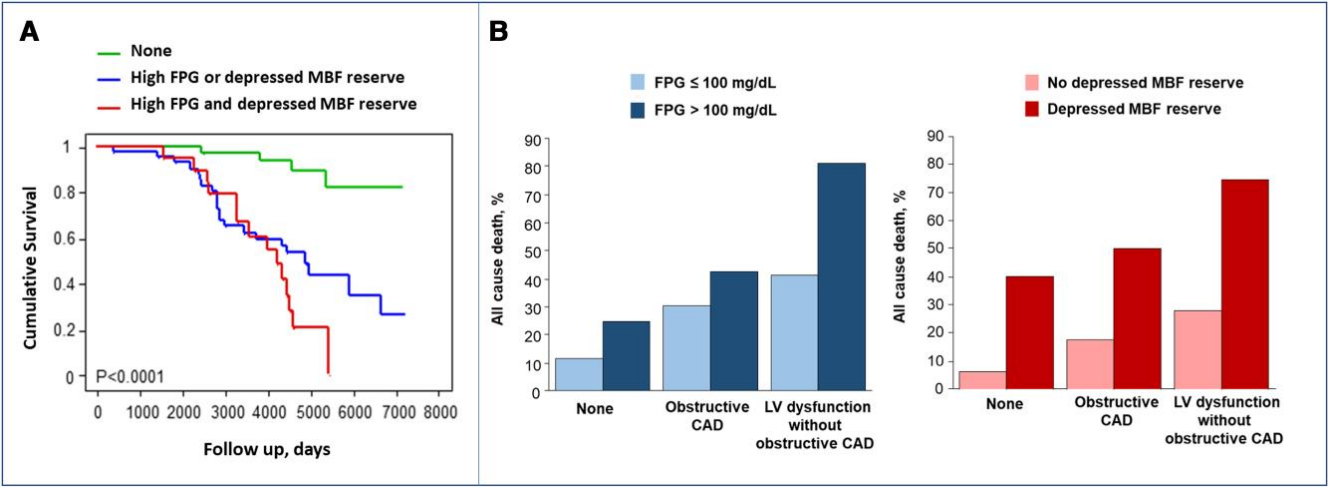
All-cause death rate in patients groups stratified for FPG or MBF reserve. (*A*) The Kaplan–Meier survival curves obtained in patients without hFPG and without depressed MBF reserve, with only one of them and with both. The combination of hFPG and depressed MBF reserve is associated with the worst prognosis. (*B*) Death rate progressively increasing across disease phenotypes, being higher in patients with hFPG (dark bars) or with MBF reserve < 2 (dark bars) in each disease group. FPG, fasting plasma glucose; MBF, Myocardial blood flow.

**Table 2:** Univariate and multivariate analysis of the association between baseline clinical characteristics, risk factors, MBF reserve, disease phenotypes, and all-cause death

	Univariate analysis		Multivariate analysis			
	HR (95% CI)	*P* value	HR (95% CI)	*P* value	HR (95% CI)	*P* value
Age	1.05 (1.01–1.09)	**0**.**018**	1.06 (1.02–1.11)	**0**.**007**	1.06 (1.02–1.12)	**0**.**007**
Sex male	1.81 (0.83–3.95)	0.136	1.20 (0.49–2.93)	0.690	1.38 (0.49–3.86)	0.546
Smoking	1.38 (0.74–2.56)	0.310	1.32 (0.64–2.73)	0.452	1.28 (0.61–2.69)	0.514
Hypercholesterolaemia	1.27 (0.60–2.67)	0.528	0.81 (0.35–1.85)	0.612	0.85 (0.34–2.12)	0.729
Hypertension	1.18 (0.63–2.18)	0.606	0.79 (0.40–1.55)	0.490	0.85 (0.42–1.72)	0.641
Obesity	0.84 (0.37–1.91)	0.681	0.65 (0.26–1.64)	0.359	0.60 (0.22–1.68)	0.318
Type 2 diabetes	1.01 (0.44–2.27)	0.993	0.77 (0.31–1.93)	0.580	0.81 (0.32–2.06)	0.720
Low HDL-C	0.85 (0.46–1.56)	0.592	0.82 (0.40–1.68)	0.592	0.85 (0.40–1.77)	0.656
High TG	1.11 (0.57–2.17)	0.767	1.31 (0.60–2.84)	0.495	1.26 (0.57–2.76)	0.567
hFPG	2.43 (1.31–4.51)	**0**.**005**	2.28 (1.09–4.79)	**0**.**029**	2.18 (1.02–4.63)	**0**.**043**
Depressed MBF reserve	4.93 (2.34–10.38)	**<0**.**001**	4.79 (2.14–10.69)	**<0**.**001**	4.47 (1.96–10.18)	**<0**.**001**
Obstructive CAD	2.34 (0.67–8.24)	0.184	—	—	0.85 (0.18–4.11)	0.836
LV dysfunction	3.40 (1.02–11.37)	**0**.**047**	—	—	1.18 (0.30–4.57)	0.816

Association of baseline clinical characteristics, risk factors, disease phenotypes, MBF reserve, and all-cause death was assessed using univariable and multivariable Cox analyses. *P* values <0.05 (bold) show statistically significant differences.

CAD, coronary artery disease; LDL-C, low-density lipoprotein cholesterol; hFPG, high fasting plasma glucose; HDL-C, high-density lipoprotein cholesterol; TG, triglycerides; MBF, myocardial blood flow.

When disease phenotypes were added to the multivariable Cox model, age (HR 1.07, 95% CI 1.02–1.12), hFPG (HR 2.18, 95% CI 1.02–4.63), and depressed MBF reserve (HR 4.47, 95% CI 1.96–10.18) remained independent predictors of death (global *χ*^2^ 37.41, *P* = 0.0004) (*Table 2*). *Figure 1B* plots the observed all-cause death rate in patients with different disease phenotypes stratified for absence/presence of hFPG or MBF reserve <2. Death rate progressively increases according to disease phenotypes and is significantly associated with hFPG (*P* = 0.049) or impaired MBF reserve (*P* = 0.012) in each patient group.

Major adverse cardiovascular events (MACE) (all-cause death and hospitalization for cardiac causes) occurred in 60 patients (58.3%). At multivariable Cox analyses, including all risk factors and disease phenotypes, age (HR 1.04, 95% CI 1.01–1.07) and depressed MBF reserve (HR 2.52, 95% CI 1.39–4.57) remained the only independent predictors of MACE (global *χ*^2^ 33.52, *P* = 0.0014).

These results expand the evidence of the strong prognostic role of globally depressed MBF reserve in heart diseases^5–7,9^ showing, at a prolonged follow-up in a high-risk population, how it stands beyond established cardiovascular risk factors and the heart disease phenotype. Our results also reveal that hFPG, among established and emerging risk factors, was the only one associated with global coronary dysfunction and able to identify patients at residual risk of overall mortality across coronary atherosclerotic and myocardial disease phenotypes.

The two findings may be linked together. There is evidence that hyperglycaemia, tracking closely with the increased burden of prediabetes, diabetes, and obesity,^1–3^ may cause important alterations in global coronary function by a range of structural and functional macrovascular and microvascular abnormalities.^6,7^

The present study suffers of multiple limitations mainly due to the retrospective design and the very prolonged follow-up. The sample size was significantly reduced by exclusion of patients with incomplete datasets. In the last 20 years, management of diabetes, impaired glucose and lipid metabolism as well as HF has significantly evolved with introduction of new agents. Accordingly, our results need to be confirmed in a larger well-defined high-risk population, receiving updated management and with a similar prolonged follow-up (>10 years). If confirmed, they may have relevant practical implications underscoring the relevance of recognizing impaired FPG and globally depressed coronary function in patients with CAD or HF to stratify residual individual mortality risk and promote targeted therapy. As a matter of fact, hFPG, in the absence of overt diabetes, is still not included in existing prognostic models and measurement of MBF reserve is not common practice. Nevertheless, these two variables might be relevant to predict evolution of CAD and HF from early stages as well as to stratify risk in patients with established disease providing potential targets for personalized management. New therapeutic strategies are promising in this context. In fact, although lifestyle management through diet and exercise is the foundation for treatment of hyperglycaemia, more recently, SGLT-2 inhibitors and GLP-1Ra have been shown to reduce cardiovascular outcomes and all-cause mortality in higher-risk individuals, with or without diabetes, with pre-existing heart diseases or multiple risk factors.^3,10^ The present results might also stimulate future research to assess whether these or other drugs able to improve glucose homeostasis might exert their protective effects in different heart diseases by targeting multiple mediators of coronary macrovascular and microvascular pathophysiology translating into an improvement of global coronary and myocardial function.^9,10^

## Consent

The authors attest they are in compliance with human studies committees of the authors’ institutions, including patient consent where appropriate.

## Data Availability

The data that support the findings of this study are available from the corresponding author upon reasonable request.

## Lead author biography

As a Cardiologist and Nuclear Imaging specialist, Dr. Neglia has been dedicated for many years to clinical and translational research in coronary artery disease, dilated cardiomyopathy and heart failure. His clinical and research interest is currently focused on the use of Multimodality Cardiovascular Imaging at his Institution, Fondazione Toscana G. Monasterio in Pisa, and within the European Association of Cardiovascular Imaging as Chair of the Research&Innovation Committee. Dr. Neglia has coordinated large European trials and registries focused on diagnosis and treatment of ischemic heart disease. He is teaching to PhD students of Translational Medicine at Scuola Sant'Anna in Pisa.
